# N-myc downstream-regulated gene 1 inhibits the proliferation of colorectal cancer through emulative antagonizing NEDD4-mediated ubiquitylation of p21

**DOI:** 10.1186/s13046-019-1476-5

**Published:** 2019-12-12

**Authors:** Sen Zhang, Chaoran Yu, Xiao Yang, Hiju Hong, Jiaoyang Lu, Wenjun Hu, Xiaohui Hao, Shuchun Li, Batuer Aikemu, Guang Yang, Zirui He, Luyang Zhang, Pei Xue, Zhenghao Cai, Junjun Ma, Lu Zang, Bo Feng, Fei Yuan, Jing Sun, Minhua Zheng

**Affiliations:** 1 Department of General Surgery, Ruijin Hospital, Shanghai Jiao Tong University School of Medicine, Shanghai, China; 2 Shanghai Minimally Invasive Surgery Center, Ruijin Hospital, Shanghai Jiao Tong University School of Medicine, Shanghai, China; 3 Shanghai Institute of Digestive Surgery, Ruijin Hospital, Shanghai Jiao Tong University School of Medicine, Shanghai, China; 4 Department of Pathology, Ruijin Hospital, Shanghai Jiao Tong University School of Medicine, Shanghai, China

**Keywords:** NDRG1, Tumor proliferation, p21, Ubiquitylation, NEDD4

## Abstract

**Background:**

N-myc downstream-regulated gene 1 (NDRG1) has been shown to play a key role in tumor metastasis. Recent studies demonstrate that NDRG1 can suppress tumor growth and is related to tumor proliferation; however, the mechanisms underlying these effects remain obscure.

**Methods:**

Immunohistochemistry (IHC) was used to detect NDRG1 and p21 protein expression in colorectal cancer tissue, and clinical significance of NDRG1 was also analyzed. CCK-8 assay, colony formation assay, flow cytometry, and xenograft model were used to assess the effect of NDRG1 on tumor proliferation in vivo and in vitro. The mechanisms underlying the effect of NDRG1 were investigated using western blotting, immunofluorescence, immunoprecipitation, and ubiquitylation assay.

**Results:**

NDRG1 was down-regulated in CRC tissues and correlated with tumor size and patient survival. NDRG1 inhibited tumor proliferation through increasing p21 expression via suppressing p21 ubiquitylation. NDRG1 and p21 had a positive correlation both in vivo and in vitro. Mechanistically, E3 ligase NEDD4 could directly interact with and target p21 for degradation. Moreover, NDRG1 could emulatively antagonize NEDD4-mediated ubiquitylation of p21, increasing p21 expression and inhibit tumor proliferation.

**Conclusion:**

Our study could fulfill potential mechanisms of the NDRG1 during tumorigenesis and metastasis, which may serve as a tumor suppressor and potential target for new therapies in human colorectal cancer.

## Background

According to the latest globally statistics released in 2018, colorectal cancer (CRC) is estimated to be the third most commonly diagnosed malignant tumor (10.2% of the total cases) and the second leading cause of cancer-related deaths (9.2% of the total cancer-related deaths) [1]. In China, the age-standardized incidence of CRC increased from 12.8 (2003) to 16.8 (2011) per 100,000 individuals [2]. Although curative surgery is still the main treatment for CRC patients, most cases are diagnosed at advanced stage, or, worse, with metastasis. The prognosis is poor, even though many novel treatment strategies, such as targeted therapy and immunotherapy have been used. Therefore, development of novel treatment targets is important to help design novel therapies [3]. In addition, there is also a great need to elucidate the molecular mechanisms of different subgroups of CRC, which can provide critical clues for the personalized treatment.

N-myc downstream regulated gene-1 (NDRG1), a member of the NDRG gene family, is pervasively expressed in epithelial tissues and is a cytoplasmic and nuclear protein [4–6]. NDRG1 has been proved to play a key role in tumor proliferation, metastasis, differentiation, cell adhesion, cell-cycle modulation, and autophagy [7–12]. Evidences have demonstrated that NDRG1 expression was significantly decreased in several malignant diseases, including gastric, colorectal, prostate, and breast cancer [13–16]. Therefore, NDRG1 is recognized as “tumor suppressor”, which can induce tumor differentiation, inhibit invasion, metastasis, and cell proliferation [13]. Besides, NDRG1 expression is closely associated with a positive survival of colon [17], prostate [18], breast [15] and pancreatic cancer patients [19]. It has been reported that locally advanced rectal cancer patients with NDRG1-positive expression could benefit from oxaliplatin-dominated chemotherapy [20]. In the previous studies, our team has elaborately revealed part of the functions and underlying mechanisms of NDRG1 during CRC progression and metastasis [10, 17, 21–24], however, there are limited studies regarding the mechanism of NDRG1 in proliferation in human solid tumors. Recent studies have shown NDRG1 could induce cancer cell G0/G1 arrest, inhibiting tumor proliferation [13, 25]. Yet, its underlying mechanism is still obscure.

Dysregulated proliferation is a notable feature of tumorigenesis. Unlike the normal cells, whose proliferation is well balanced by growth and antigrowth signals, the tumor cells have their own growth signals, and their proliferation is precisely regulated by cell cycle regulators [26]. p21, the inhibitor of the cyclin-dependent kinase and effector of the p53 tumor suppressor, is indispensable for cell-cycle progression, which can arrest most G1-phase in response to various stimuli [27]. Various genes could promote the tumor proliferation and progression by inhibiting p21 [28, 29]. In addition, there are studies suggesting that loss expression and/or function of p21 may contribute to tumorigenesis and metastasis [27, 30].

Therefore, based on the functions of NDRG1 and p21 in diseases, there may be interactions between NDRG1 and p21. In this study, we demonstrated that NDRG1 could inhibit tumor proliferation through increasing p21 protein expression in vivo and in vitro*.*

## Methods

### Tissue specimens and immunochemistry (IHC)

A total of 89 CRC cases were recruited from Ruijin Hospital (Shanghai, China) in accordance with the guidelines set by the Ethical Committee of Ruijin Hospital. All cases got the written, informed consent before the study. Staging of CRC was performed according to the UICC guideline (8th Edition). The cohort of 89 tumor tissues and paired normal colonic tissues were fixed with formaldehyde and embedded with paraffin. Tissue array was constructed for further immunohistological assay. The staining score of each tissue was calculated based on the widely used German semi-quantitative scoring system by three independent pathologists as described previously [22, 31, 32], score > 3 was considered positive expression.

### Cell culture and transfection

The p53-wt HCT116, the p53-mutant SW1116 colon cancer cell lines and HEK 293 T cells were obtained from the American Type Culture Collection (ATCC, Manassas, VA). The NDRG1 overexpression and knockdown clones were established as described previously [23]. All cells were cultured under a humidified atmosphere containing 5% CO_2_ at 37 °C. The plasmid vector containing NEDD4, Flag-p21 and HA-Ubiquitin were constructed by Genechem Inc. (Shanghai, China). Lentivirus vectors containing Flag-p21 were constructed by Genechem Inc. (Shanghai, China). For a transient transfection, cells were seeded in a 6-well culture plate 24 h before transfection, then cells were transfected with corresponding si-RNA or vector, using Lipofectamine 3000 (Invitrogen, Carlsbad, CA) following the manufacturer’s protocol. Sequences of siRNA used were as follows: p21: sense-1 5′-GAUGGAACUUCGACUUUGUTT-3′, antisense-1 5′-ACAAAGUCGAAGUUCCAUCTT-3′; sense-2 5′-CCUCUGGCAUUAGAAUUAUTT-3′, antisense-2 5′-AUAAUUCUAAUGCCAGAGGTT-3′; sense-3 5′-CAGGCGGUUAUGAAAUUCATT-3′, antisense-3 5′-UGAAUUUCAUAACCGCCUGTT-3′. NEDD4: sense-1 5′-GGAUGUUCCAACUCAUCUUTT-3′, antisense-1 5′-AAGAUGAGUUGGAACAUCCTT-3′; sense-2 5′-CCAAGAAGUCACAAAUCAATT-3′, antisense-2 5′-UUGAUUUGUGACUUCUUGGTT-3′; sense-3 5′-GCACAUCUCGGGUGCCUAUTT-3′, antisense-3 5′-AUAGGCACCCGAGAUGUGCTT-3′.

### Colony formation and CCK-8 assay

For colony formation, after dissociated into single cell, 500 tumor cells (HCT116) or 1000 tumor cells (SW1116) were seeded in six-well plate, which were cultured in an incubator until visible cloning appeared. Then these six-wells plates were washed, fixed with 4% formaldehyde, stained with crystal violet dye, calculated and analyzed.

For CCK-8 assay, six repeats of 1500 cells were seeded in 96-well plate and cultured in each group. The growth rates of the cells were determined at 24, 48, 72, 96 and 120 h using Cell Counting Kit-8 (Dojindo Molecular Technologies, Rockville, Japan). The absorbance was measured at 450 nm using a model 3550 microplate reader (Bio-Rad Laboratories, Inc., Hercules, CA, USA).

### Western blot analysis

Cells were washed with PBS and then lysed with RIPA buffer. Insoluble materials were removed by centrifugation at 12,000 rpm for 15 min at 4 °C. Equal amounts of protein were separated using 10% SDS-PAGE, transferred to PVDF, and probed with the appropriate antibodies as indicated. Immunoreactive bands were visualized using an ECL kit (Amersham Biosciences, Piscataway, New Jersey, USA). The extraction of proteins from nuclear and cytoplasmic fractions were performed using Subcellular Protein Fractionation Kit from Thermo Scientific (78840; Waltham, MA, USA). Primary antibodies including: NDRG1 (Rabbit, catalog number ab124689) from Abcam; p21 (Rabbit, catalog number 2947) from Cell Signaling Technology; p21 (Mouse, catalog number ab80633) from Abcam; NEDD4 (Rabbit, catalog number 2740) from Cell Signaling Technology; NEDD4 (Rabbit, catalog number 21698-1-AP) from Proteintech; Flag (Rabbit, catalog number 14793) and Histone H3 (Rabbit, catalog number 4499) from Cell Signaling Technology. The secondary antibodies implemented include horseradish peroxidase-conjugated anti-goat (catalog number A5420), anti-rabbit (catalog number A6154), and anti-mouse (catalog number A4416) antibodies from Sigma-Aldrich.

### RNA extraction and qPCR for mRNA expression analysis

RNA extraction and qPCR analysis were performed as described previously [22, 33]. The primer sequences used for analysis are listed as follows: NDRG1 (5′-CTGCACCTGTTCATCAATGC-3′ and 5′-AGAGAAGTGACGCTGGAACC-3′); p21 (5′-AGGTGGACCTGGAGACTC-3′ and 5′-CGGCGTTTGGAGTGGTAG-3′); GAPDH (5′-TTCAACAGCAACTCCCACTCTT-3′ and 5′-TGGTCCAGGGTTTCTTACTCC-3′).

### Immunoprecipitation and ubiquitylation assay

Cells were washed twice with ice-cold PBS and lysed by RIPA buffer containing protease inhibitors (Roche Diagnostics, Basel, Switzerland). Protein (300 mg) was incubated with specific antibody overnight at 4 °C. This mixture was added to 30 ul of beads (Protein A/G PLUS-Agarose, sc-2003) from Santa Cruz Biotechnology (Santa Cruz, CA) and incubated for 4 h at 4 °C. Then washed by ice-cold PBS, resuspended in loading buffer, and incubated over 90 °C for 10 min. The supernatant was separated on a 10% Bis-Tris gel. Then the mixture was detected by western blots.

UbiBrowser Database was used to screen possible E3 ligases related to NDRG1 or p21 (http://ubibrowser.ncpsb.org/). The co-expression analysis of NDRG1 and p21 in GSE33114 and GSE37892 profiles was performed via the R2 Genomics Analysis and Visualization Platform (http://r2.amc.nl). The co-expression analysis of NDRG1 and p21 in the cancer genomic atlas (TCGA) was performed in The Gene Expression Profiling Interactive Analysis (GEPIA, http://gepia.cancer-pku.cn/index.html).

Ubiquitylation assay was performed as described previously. The cells were treated with Mg132 and then lysed by SDS-free RIPA buffer and immunoprecipitated with primary antibody followed by protein A/G plus agarose. Then, the supernatant was detected by immunoblotting using ubiquitin antibody (FK2, Enzo Life Sciences, New York, NY, USA).

### Immunofluorescence

As described previously [23], cells were incubated with the relative primary antibody overnight at 4 °C, followed by incubation with fluorescent secondary antibody for 2 h at room temperature. After final washes with phosphate-buffered saline (PBS), the coverslips were mounted using an antifade mounting solution containing 4,6-diamidino-2-phenylindole (DAPI; P36935, Invitrogen). Images were acquired on the confocal microscope (Carl Zeiss). Primary antibody:anti-rabbit p21 (catalog number 2947) from Cell Signaling Technology; anti-mouse p21 (catalog number ab80633) from Abcam for Immunofluorescence double-staining analysis; anti-rabbit NEDD4 (catalog number 2740) from Cell Signaling Technology.

### Xenograft model

Mice were cultivated under standard conditions following institutional guidelines. A total of 5 × 10^6^ HCT116 cells (NDRG1 overexpression, knockdown and corresponding controls) or SW1116 cells (sh-Control, SH-NDRG1 and SH-NDRG1/p21) were injected subcutaneously into nude mice (male BALB/c nu/nu nude mice, 4-week-old). Tumor size was measured every 7 days. Tumor volume (V) was determined by measuring the length and width of the tumor and using the formula V = (width*width*length) / 2. Twenty-eight days after injection, all the mice were sacrificed and then tumor grafts were excised, fixed with formalin and embedded with paraffin for further immunohistochemistry research.

### Statistical analysis

IBM SPSS Statistical software (version 19.0) was utilized for statistical analysis. Differences were compared using a two-tailed Student *t* test. Differences with a *P* value < 0.05 were considered as statistically significant.

## Results

### NDRG1 was down-regulated in human CRC tissues and was positively related to p21 expression as well as prognosis

**Fig. 1:**
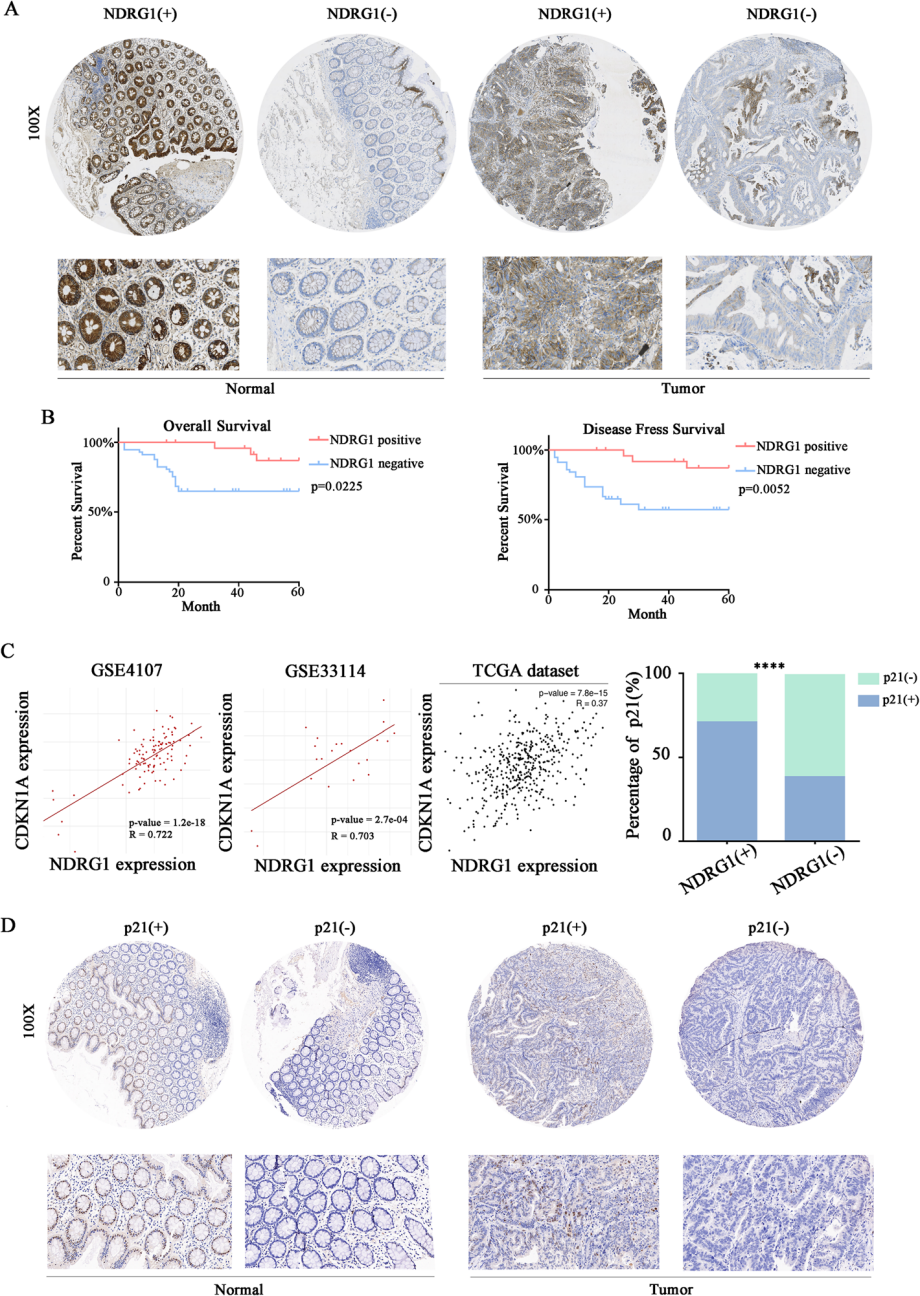
Expression of NDRG1 and its clinical significance in CRC patients. **a** NDRG1 expression in tumor and paired normal tissues was evaluated by IHC with tissue array. **b** Overall Survival and Disease Free Survival of patients with CRC having NDRG1(+) and NDRG1(−) expression. **c** Correlation analysis of NDRG1 and p21 in TCGA colorectal cancer datasets and our cohort specimen. **d** p21 expression in tumor and paired normal tissues. ****p < 0.0001

A tissue array containing 89 pairs of cancer and matched normal tissues was used to examine the expression of NDRG1 by Immunohistochemistry assay (IHC). Results showed that NDRG1 expression was significantly lower in CRC tissues compared to their corresponding non-tumorous tissues (Fig. 1a, *p* < 0.0001). Further analysis demonstrated that NDRG1 was positively stained (+) in 59.6% (53/89) of normal tissues, whereas only 29.2% (26/89) of carcinoma cases were positive to NDRG1 stain (*p* <  0.0001). To investigate the clinical significance of NDRG1 in CRC, the correlation between NDRG1 expression and clinicopathological variables was analyzed. The data showed a negative correlation between NDRG1 expression and local invasion, as well as lymphatic metastasis (Table 1). Besides, analysis showed that there was a higher proportion of negative expression of NDRG1 when tumor size was > 3 cm (15 and 48 cases of NDRG1 negative expression for size ≤3 cm and size > 3 cm, respectively, *p* <  0.001), indicating that NDRG1 might be related to tumor proliferation. As for Overall Survival (OS) and Disease-Free Survival (DFS), patients in the NDRG1-negative group (−) had a significantly poorer prognosis than those in the NDRG1-positive group (+) (Fig. 1b).

**Table 1:** The relationship betwee NDRG1 expression and clinical features in this study

Variable	case	NDRG1 expresssion		*P* value
		Positive	Negative	
Tissue				< 0.0001
Normal	89	53	36	
Cancer	89	26	63	
Age				0.068
≤ 60	39	12	27	
> 60	50	25	25	
Gender				0.399
Male	48	18	30	
Femal	41	19	22	
Lymphatic invasion				0.009
N0	48	26	22	
N1 + N2	41	11	30	
Local invasion				0.019
T1 + T2	47	25	22	
T3 + T4	42	12	30	
TNM stage				0.087
I + II	37	18	19	
III + IV	52	16	36	
Tumor Diameter				< 0.001
≤ 3 cm	31	16	15	
> 3 cm	58	10	48	

p21, a key regulator of tumor proliferation [34], can arrest most G1-phase in response to various stimuli [27], which is indispensable for cell-cycle progression. Various genes could promote the tumor proliferation and progression by inhibiting p21 [28, 29]. Moreover, it’s been reported that NDRG1 could up-regulate p21 protein expression.

To explore the expression and potential relationship between NDRG1 and p21 in CRC development, we first evaluated their mRNA expression by querying the public available database. The correlation of *NDRG1* and *CDKN1A* was analyzed in three colorectal cancer datasets (GSE33114, GSE4107, and TCGA). The results showed an obvious positive relationship between *NDRG1* and *CDKN1A* in GSE33114 (r: 0.722, *p* <  0.0001), GSE37892 (r: 0.703, p <  0.0001), and TCGA (r: 0.37, *p* ≤ 0.0001) (Fig. 1c).

In accordance with the dataset-based analysis, the IHC analysis of 89 pairs of cancer and matched normal tissue showed that p21 expression was well correlated with NDRG1 expression (Fig. 1c and d). Specifically, 56 cases (70.9%) out of 79 NDRG1 positive cases were p21 positive, while among 99 NDRG1 negative cases, 60 cases (60.6%) were p21 negative. NDRG1 was positively correlated with p21 expression in this cohort.

Therefore, NDRG1 was down-regulated in human CRC tissues and was positively related with p21 expression, as well as prognosis.

### NDRG1 up-regulated p21 protein expression in CRC cells in vitro

**Fig. 2:**
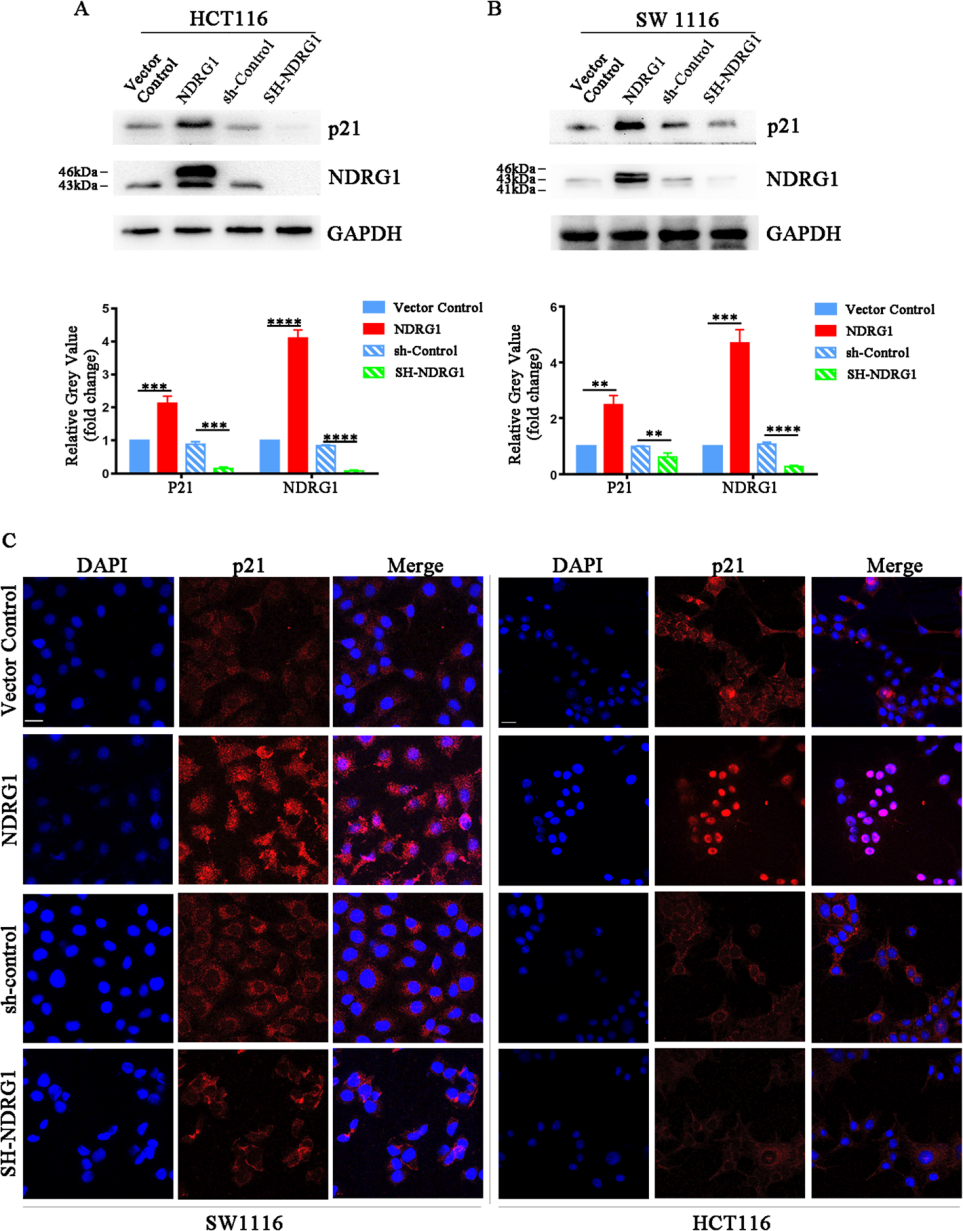
NDRG1 could increase p21 expression in colorectal cancer. **a** and (**b**) Changes in p21 expression after NDRG1 overexpression and knockdown in HCT116 and SW1116 cell lines. GAPDH was used as loading control for whole cell lysates. Densitometry represents the expression of the proteins relative to GAPDH. **c** Representative images of p21 expression by immunofluorescence staining (red: p21; blue: nuclear (DAPI), scale bar: 50 μm and 20 μm for SW1116 and HCT116, respectively). Error bar represents the mean ± SD of three independent experiments. *p < 0.05, **p < 0.01, ***p < 0.001, ****p < 0.0001

As previously reported [10, 22], we established HCT116 and SW1116 CRC cell lines that stably overexpressed exogenous NDRG1. Besides, NDRG1-knockdown models were also generated in these cell lines. We analyzed p21 protein expression after NDRG1 overexpression/knockdown. As immunoblots showed (Fig. 2a and b), p21 protein expression was significantly increased (1.1–1.8-fold, *p* < 0.01) after NDRG1 overexpression, while NDRG1-knockdown obviously decreased the expression (36–89%, *p* < 0.01) of p21 in HCT116 and SW1116 cell lines. Besides, we checked the intensity of p21 by immunofluorescence staining. In agreements with the immunoblot results, immunofluorescence showed that the staining intensity of p21 was obviously increased and decreased in response to NDRG1 overexpression and knockdown, respectively (Fig. 2c). Besides, the results showed that there was more p21 accumulation in the nucleus induced by NDRG1 overexpression, a crucial phenomenon for the function of p21.

Expression of p21 protein was also tested in the cytoplasmic and nuclear fractions of cells. The subcellular fractionation assays indicted that there was a significant accumulation of p21 in both the nucleus and cytoplasm after NDRG1 was overexpressed. Whereas, when NDRG1 was silenced, p21 markedly decreased in both fractions in HCT116 and SW1116 cell lines (Additional file 2: Figure S1A). We also analyzed p21 mRNA levels using qPCR, the results indicated that there were not significant changes in p21 mRNA levels after NDRG1 was overexpressed/silenced in both HCT116 and SW1116 cell lines (Additional file 2: Figure S1B).

### NDRG1 inhibited the CRC cell proliferation by increasing p21 expression

**Fig. 3:**
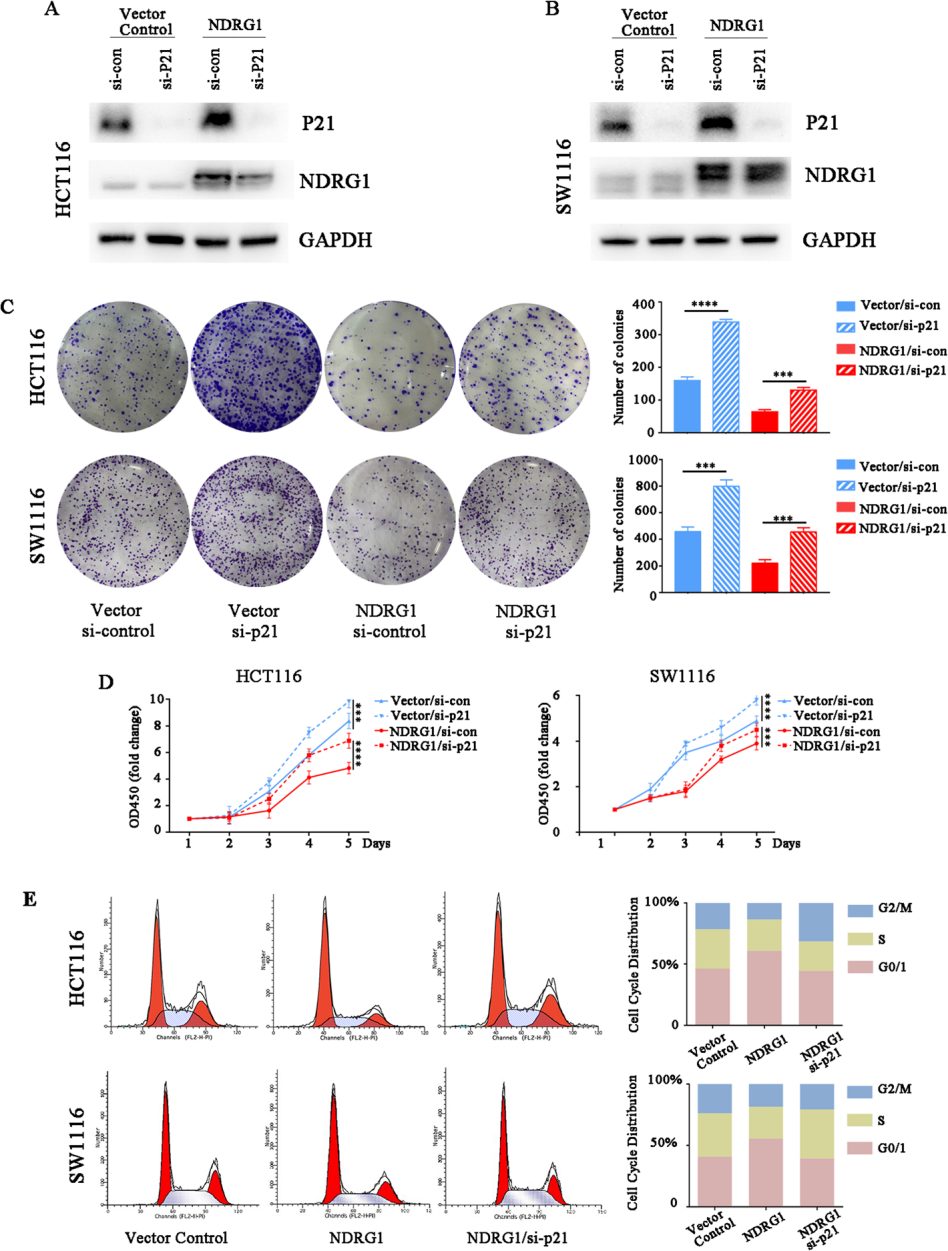
NDRG1 inhibited cell proliferation through increasing p21 expression in colorectal cancer. **a** and (**b**) Endogenous p21 was knocked down after transfection with si-RNA-p21. Colony formation assay (**c**), CCK-8 assay (**d**), and cell cycle analysis (**e**) were performed to demonstrate that p21 knockdown could rescue the inhibitory effect on CRC cell proliferation induced by NDRG1 overexpression. Error bar represents the mean ± SD of three independent experiments. *p < 0.05, **p < 0.01, ***p < 0.001, ****p < 0.0001

Both NDRG1 and p21 are tumor suppressors, combined with the results above, thus, we hypothesized that NDRG1 might inhibit tumor proliferation via up-regulating p21 expression. After NDRG1 overexpression, we explored the effect of p21 on CRC cell proliferation. Colony formation assay showed that NDRG1 overexpression alone clearly inhibited the cell proliferation, while silencing p21 alone by si-RNA significantly increased proliferation. Interestingly, the inhibited proliferation by NDRG1 overexpression was almost restored after the p21 was silenced in both HCT116 and SW1116 cell lines (Fig. 3a, b, and c). CCK-8 assay also confirmed that loss of p21 expression could rescue the inhibitory effect of NDRG1 (Fig. 3d). Cell cycle analysis indicated that there was clearly more G0/G1 phase cell population in the NDRG1 overexpression group in compared to the relative control group (60.70% vs. 46.23% in HCT116 and 55.48% vs. 40.71% in SW1116), suggesting that NDRG1 could induce G0/G1 arrest in CRC cell lines. This inhibitory phenomenon was obviously restored after p21 expression was silenced by si-RNA (Fig. 3e), indicating that p21 played a key role in inhibiting proliferation induced by NDRG1.

Therefore, the results we presented herein indicated that NDRG1 could inhibit CRC cell proliferation by increasing p21 expression.

### NDRG1 could suppress the p21 ubiquitylation and degradation in vitro

**Fig. 4:**
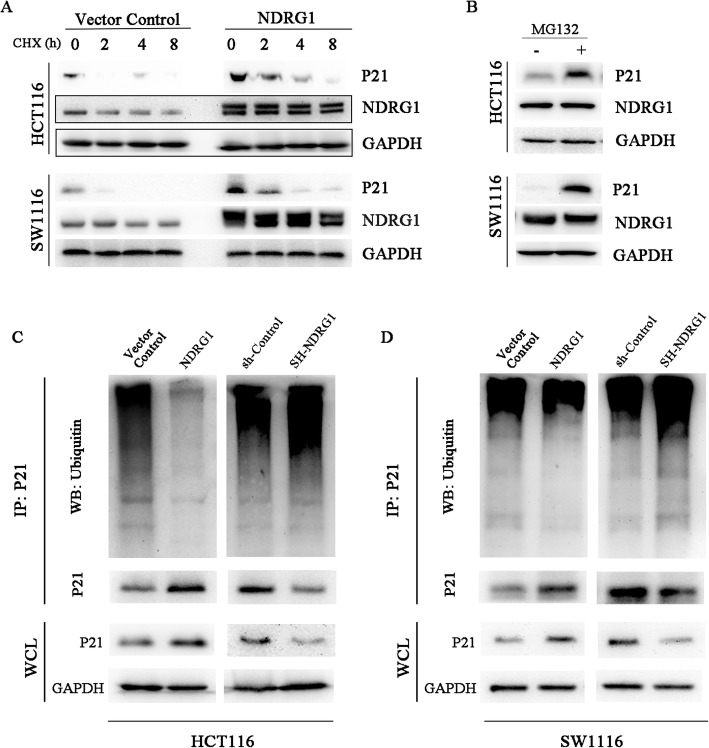
NDRG1 overexpression suppressed p21 ubiquitylation. **a** To examine the influence of NDRG1 overexpression on p21 stability, translation of the cells was arrested by treatment with cycloheximide (CHX). At various times after CHX addition, protein was extracted, and then total p21 was investigated using western blot analysis. GAPDH was used as an internal control. **b** The expression of p21 was examined after treatment with MG132, an inhibitor of the ubiquitin-proteasome system. **c** and **d** Western blots with anti-ubiquitin antibody showed p21 ubiquitylation level after NDRG1 overexpression and knockdown in both HCT116 and SW1116 cell lines

To further determine the underlying mechanism by which NDRG1 could increase p21 expression, we compared the stability of p21 in the NDRG1 overexpression and their respective control cell lines. Translation of the cells was arrested by treatment with 50 μg/mL cycloheximide (CHX). At various times after CHX treatment, aliquots of cells were removed, protein was extracted, and p21 protein in these extracts was quantified by immunoblotting analysis. As shown in Fig. 4a, NDRG1 overexpression obviously increased p21 protein stability, suggesting that NDRG1 might up-regulate the p21 expression by increasing its stability. Ubiquitylation is one of the key post-translational modifications, closely related to the protein stability. Previous studies demonstrated that p21 could go through ubiquitin-mediated degradation induced by ubiquitin cascade. In this study, we showed that p21 protein level markedly increased after proteasomal inhibitor Mg132 treatment (Fig. 4b), indicating that p21 was mainly degraded in a proteasomal manner, and the ubiquitin-proteasome system was involved in controlling p21 stability. We tested if NDRG1 could interfere with p21 protein ubiquitylation. Immunoprecipitation results showed that NDRG1 overexpression inhibited p21 protein ubiquitylation, whereas NDRG1 knockdown clearly enhanced its ubiquitylation in both HCT116 and SW1116 cell lines (Fig. 4c and d).

Taken together, these results suggested that NDRG1 could increase p21 protein expression through suppressing p21 ubiquitylation and degradation.

### NEDD4 might serve as an E3 ubiquitin ligase targeting p21 degradation

**Fig. 5:**
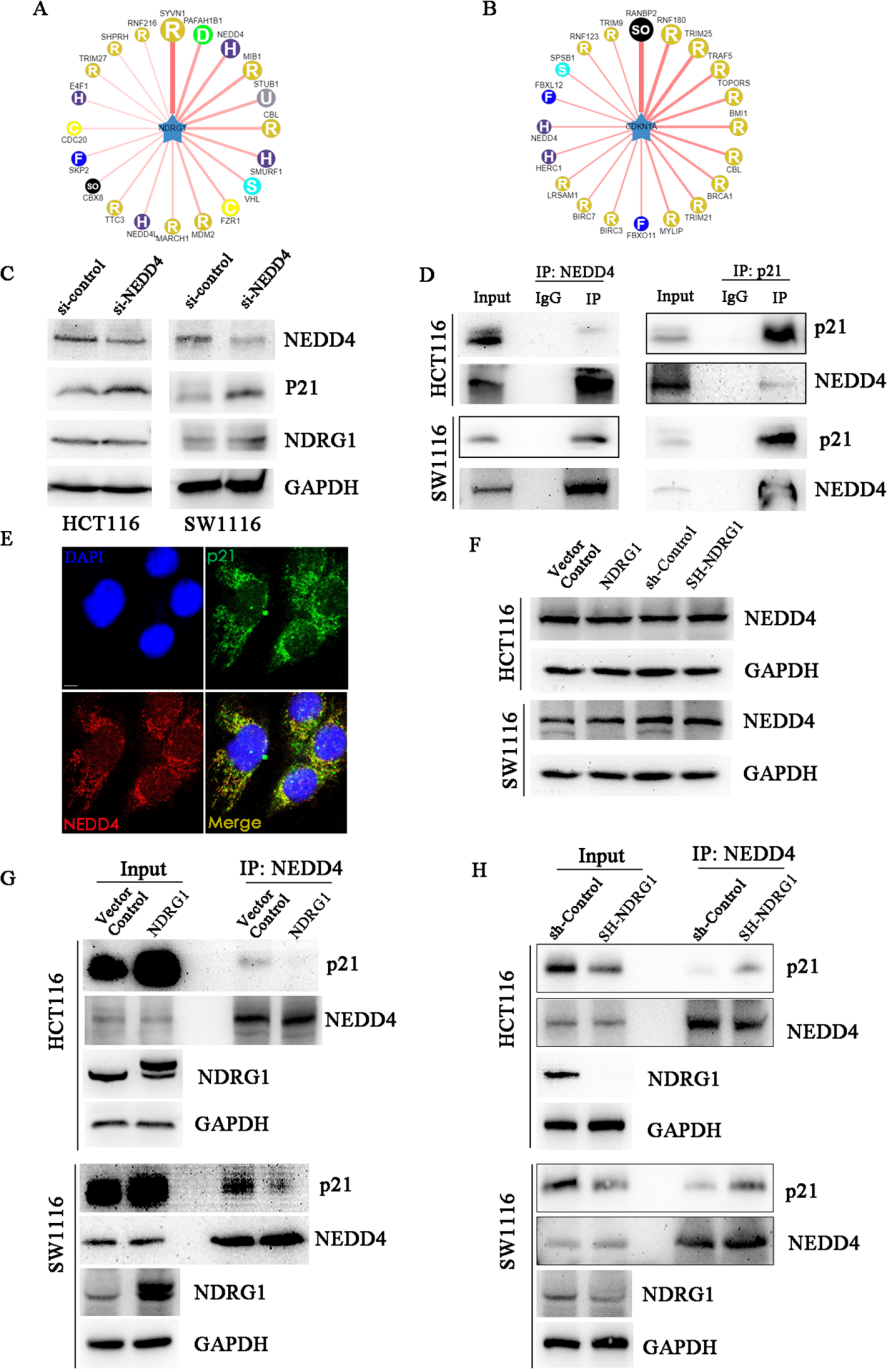
NDRG1 could emulatively antagonize NEDD4-mediated ubiquitylation of p21. **a** and (**b**) Using UbiBrowser Database (http://ubibrowser.ncpsb.org/), we screened possible E3 ligases related to NDRG1 or p21. **c** p21 expression increased after NEDD4 knockdown by si-RNA-NEDD4. **d** Co-immunoprecipitation was performed to examine whether p21 interacted with NEDD4 in both HCT116 and SW1116 cell lines. **e** Immunofluorescence co-localization assay was used to further confirm that p21 interacted with NEDD4 (red: NEDD4, green: p21, blue: DAPI, scale bar: 5 μm). **f** NEDD4 expression was not influenced by NDRG1 expression. **g** and (**h**) Co-immunoprecipitation was used to detect the changes in combination between p21 and NEDD4 after NDRG1 overexpression and knockdown

It is still not clear how NDRG1 regulates p21 ubiquitylation. E3 ligase is responsible for the ubiquitylation-induced protein degradation. As described above, p21 can be targeted by many E3 ligase. Therefore, we screened possible E3 ligases related to NDRG1 and/or p21 using UbiBrowser Database. Results showed that there were 29 and 211 possible proteins, acting as E3 ligases to target NDRG1 and p21, respectively (Fig. 5a and b, Additional file 1: Table S1). Among them, there were 20 overlapped protein which could simultaneously interact with both NDRG1 and p21 (Additional file 1: Table S1). It has been reported that NEDD4 is important for the function of NDRG1-related ubiquitylation [35]. Thus, we selected NEDD4 for further study as a potential dual E3 ligase of NDRG1/p21. To prove that p21 was the target of NEDD4, we investigated whether p21 expression was obviously up-regulated by NEDD4 si-RNA in both HCT116 and SW1116 cell lines (Fig. 5c). Furthermore, endogenous NEDD4 was immunoprecipitated from the lysates using its specific antibody, and we discovered the positive expression of co-immunoprecipitated p21 in this protein complex. Moreover, using p21 antibody to perform co-IP assay, results showed endogenous NEDD4 was also able to bind endogenous p21, which proved that p21 and NEDD4 could interact with each other in the cellular environment (Fig. 5d). In accordance with this, immunofluorescence double staining also confirmed their co-localization with a clear merge of p21 (green) and NEDD4 (red) into yellow signal (Fig. 5e).

To further confirm the relationship between p21 and NEDD4, we overexpressed both Flag-p21 and NEDD4 in HEK 293 T cell line (293 T Flag-p21/NEDD4). When Flag-p21 was immunoprecipitated using anti-flag antibody, NEDD4 could be detected in this protein complex and vice versa. Besides, NEDD4 overexpression could decrease p21 protein levels (Additional file 3: Figure S2A and 2B). To test whether NEDD4 could ubiquitinate p21, we overexpressed HA-ubiquitin, Flag-p21 or NEDD4 in 293 T cell line. p21 ubiquitylation obviously increased after NEDD4 was overexpressed (Additional file 3: Figure S2C). These results above implicated that NEDD4 could interact with p21, increase p21 ubiquitylation and target p21 protein for degradation.

### NDRG1 emulatively antagonized NEDD4-mediated ubiquitylation of p21

We found that NDRG1 overexpression did not affect the NEDD4 expression, neither did NDRG1 knockdown (Fig. 5f). Thus, it was reasonable to speculate that NDRG1 might interfere with the interaction between NEDD4 and p21. In co-immunoprecipitation experiments, we used NEDD4 antibody to specifically immunoprecipitate NEDD4 protein complex in the NDRG1 overexpression group, knockdown group, and their relative control groups. We detected the level of p21 protein expression in this complex to analyze the combination capacity. The co-IP results suggested that same amounts of NEDD4 interacted with less p21 after NDRG1 overexpression in HCT116 cell line, while NDRG1 knockdown induced more combination between NDRG1 and p21. Similar results were obtained in SW1116 cell line (Fig. 5g and h). Taken together, our results suggested that NDRG1 might emulatively antagonize NEDD4-mediated ubiquitylation of p21.

### NDRG1 suppressed tumor growth and had a positive correlation with p21 in vivo

**Fig. 6:**
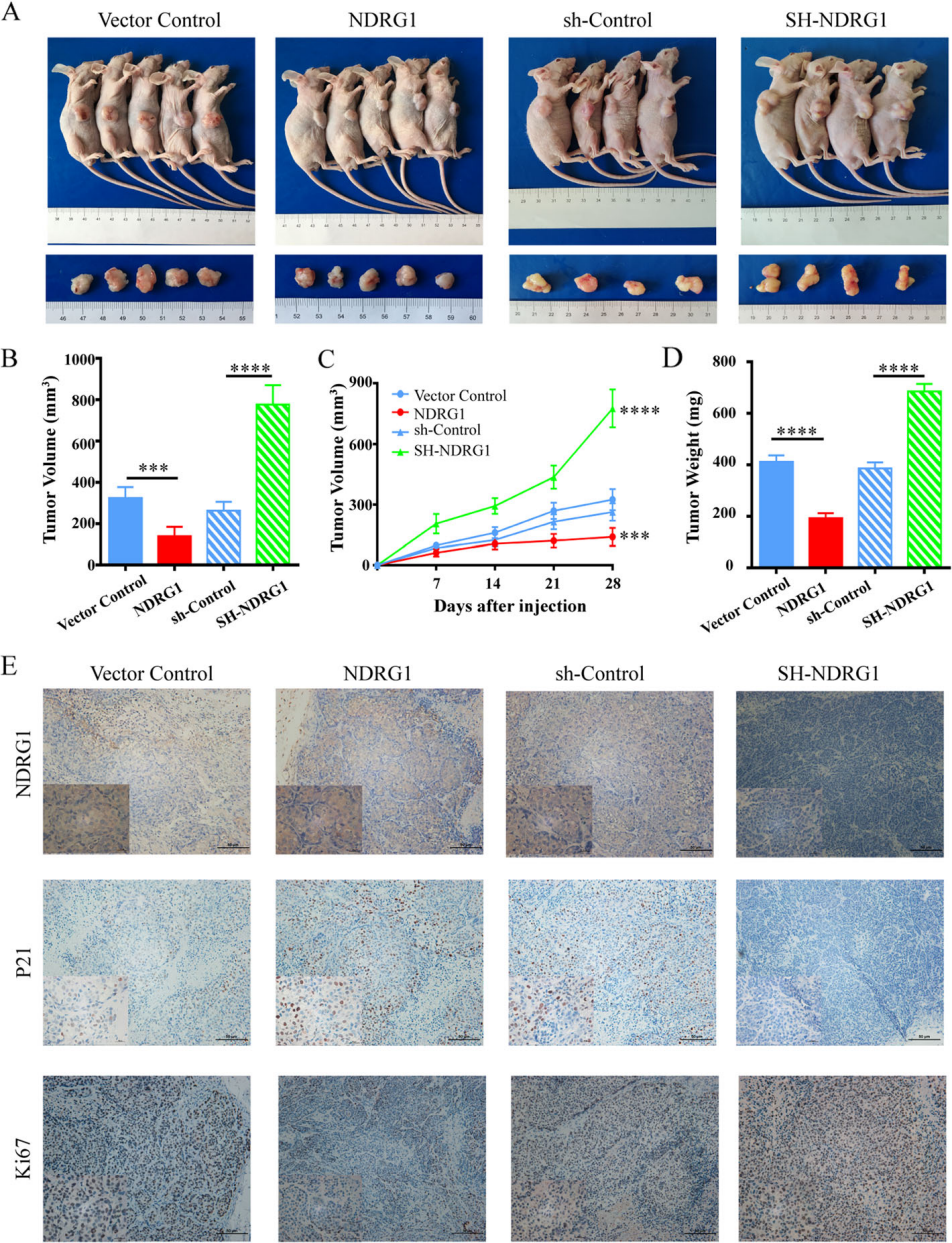
NDRG1 overexpression inhibited tumor growth in vivo. **a** Morphological observation of tumors formed after injection of nude mice with HCT116 cell lines that with NDRG1 overexpression or silencing. **b** Average volumes of xenografts, (**c**) growth curves of tumors, and (**d**) weight of tumors taken from nude mice after 30 days of growth. **e** The representative IHC staining images of NDRG1, p21, and Ki-67 in tumor xenografts. Error bar represents the mean ± SD of four or five independent experiments. ***p < 0.001, ****p < 0.0001

Based on the in vitro findings, we examined the effect of NDRG1 on tumor growth and validated its correlation with p21 in xenograft models. HCT116 NDRG1 overexpression, SH-NDRG1 and their relative control cells were subcutaneously injected into flank of nude mice (Fig. 6a). Not surprisingly, the tumor xenografts grew significantly smaller in the NDRG1 overexpression group than those in the Vector-control group (139.8 ± 19.9 mm^3^ vs. 324.6 ± 23.6 mm^3^, *p* < 0.001), while the xenografts grew significantly bigger in the SH-NDRG1 group (776.3 ± 46.5 mm^3^ vs 262.5 ± 21.8 mm^3^, *p* < 0.0001) (Fig. 6b and c). The average tumor weight also showed similar trends: 192.4 ± 8.79 mg vs. 412.6 ± 11.24 mg (p < 0.0001) for NDRG1 and Vector group, 685.0 ± 14.4 mg vs. 386.2 ± 12.2 mg for SH-NDRG1 and sh-Control group (Fig. 6d). The expression of NDRG1, p21, and Ki-67 were further detected using IHC. NDRG1 overexpression induced obvious p21 expression in xenograft model, while p21 expression was almost absent in the NDRG1 knockdown group, confirming that NDRG1 and p21 expressions had a positive correlation (Fig. 6e). Furthermore, the expression of Ki-67 obviously decreased in the NDRG1 overexpression group, and increased in the SH-NDRG1 group, compared to their relative control groups (Additional file 4: Figure S3), suggesting that NDRG1 significantly inhibited the tumor growth via activating p21 function.

**Fig. 7:**
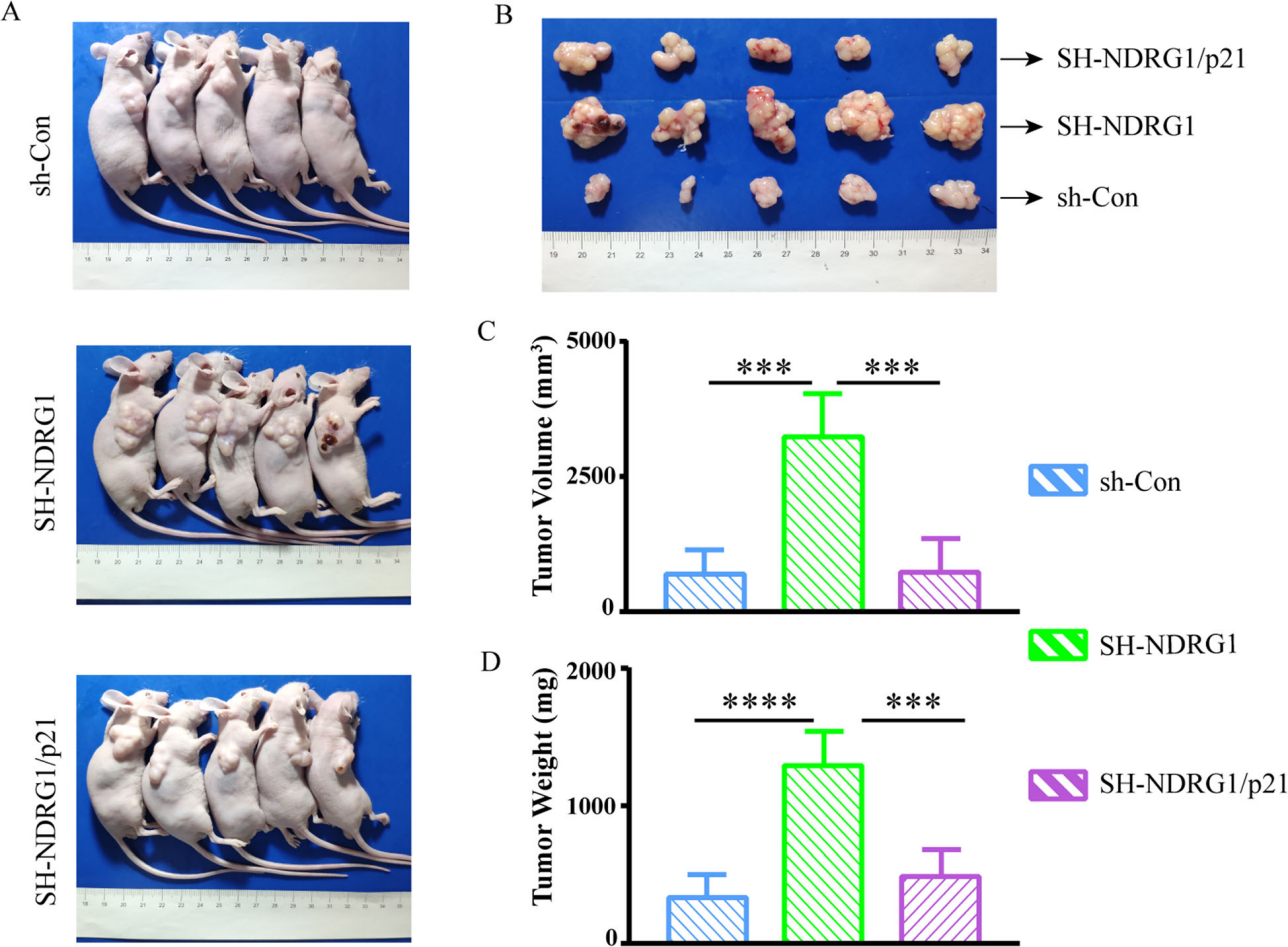
p21 could reverse the increased tumor growth induced by NDRG1 silencing in vivo. **a** and (**b**) Morphological observation of xenografts in sh-Control, SH-NDRG1, and SH-NDRG1/p21 groups. **c** Tumor volume and (**d**) Tumor weight were quantified in three groups. Error bar represents the mean ± SD of five independent experiments. ***p < 0.001, ****p < 0.0001

To further determine whether p21 was responsible for the changes in tumor growth regulated by NDRG1, we constructed stably transfected SW1116 cell lines with p21 overexpression after NDRG1 was knocked down (SH-NDRG1/p21). The sh-Control, SH-NDRG1, and SH-NDRG1/p21 cells were subcutaneously injected into the flank of nude mice. Similar to HCT116 cells, when NDRG1 was silenced in SW1116 cells, the tumor weight and volume dramatically increased compared to the relative control group (1292 ± 110.6 vs. 329.4 ± 72.72 mg, p < 0.0001) and (3247 ± 353.2 vs. 690.4 ± 201.6 mm^3^, p < 0.001) (Fig. 7). However, p21 re-expression significantly inhibited the tumor weight and volume in the SH-NDRG1/p21 group compared to the ones in the SH-NDRG1 group: (483.1 ± 88.21 vs. 1292 ± 110.6 mg, p < 0.001 and 732.2 ± 283.9 vs. 3247 ± 353.2 mm^3^, p < 0.001). All these results above indicated that p21 overexpression could attenuate the tumor growth induced by NDRG1 silencing.

## Discussion

In this study, we demonstrated that NDRG1 could inhibit tumor growth through increasing p21 protein expression. Our results indicated that NDRG1 overexpression stabilized p21 by decreasing its ubiquitylation, whereas NDRG1 silencing inhibited p21 expression by increasing ubiquitylation. NEDD4 was identified as a potential E3 ligase, which could target p21 for degradation. NDRG1 could emulatively antagonize NEDD4-mediated ubiquitylation of p21.

NDRG1, also known as Cap43, RTP, RIT42, and Drg-1 [25], normally is present in cells originating from human epithelial tissues and plays an important role in keratinocyte differentiation, attenuating hypoxic injury, myelin sheath maintenance, and cell cycle regulation [36–39]. Since the first time NDRG1 was recognized as metastasis suppressor in colon cancer [40], accumulating evidences have demonstrated that NDRG1 was down-regulated in breast, prostate, pancreatic, and colorectal cancers [14–16, 18], showing anti-tumor and anti-metastatic functions of NDRG1. However, its function in tumor development and progression still remains controversial, even in the same cancerous disease, such as hepatocellular carcinoma (HCC). Some studies reported that NDRG1 could promote HCC tumor growth, metastasis, and malignancy [41–43], while others showed that NDRG1 expression was lower in HCC tumor tissues than in normal tissues and inhibited tumor growth [25, 44]. In our previous studies, we have elaborately revealed the possible mechanisms of and related pathways of NDRG1 in inhibition of tumor metastasis [10, 17, 21–23]. Recent studies also showed that NDRG1 could inhibit proliferation of gastric cancer, oral squamous cell carcinoma (OSCC) and hepatic tumors and it was associated with good patient survival [13, 25, 45].

In agreement with previous findings, herein, we proved that NDRG1 could suppress the proliferation of CRC cell lines in vivo and in vitro. By analyzing the NDRG1 expression of 89 paired CRC samples using IHC, we showed that NDRG1 expression decreased in tumor tissues. There was a higher proportion of negative NDRG1 expression when tumor diameter > 3 cm. Tumor volume is correlated with proliferative abilities of tumor, suggesting that NDRG1 may be capable of modulation of the proliferative abilities of tumor. Moreover, xenograft models proved that NDRG1 overexpression significantly suppressed tumor growth, while NDRG1 knockdown increased growth in vivo. Ki-67 is commonly used as a proliferation marker, which is absent in G0 phase and is expressed throughout in G1, S, and G2/M phase of cell cycle [25]. The IHC analysis of xenografts showed that positive Ki-67 was more present in the NDRG1 knockdown group, suggesting that more tumor cells tended to be in the G0 (or G0/G1) phase, while Ki-67 expression was much lower in the NDRG1 overexpression group, indicating that NDRG1 was closely related to tumor proliferation.

p21 is an universal cell-cycle inhibitor, controlled by p53 and/or p53-independent pathways, closely related to tumor proliferation by inducing G0/G1 arrest [46]. In this study, NDRG1 promoted the expression of p21 protein in both HCT116 (p53-wt) and SW1116 (p53-mutant) cell lines, suggesting NDRG1 might regulated p21 in a p53-independent manner. Besides, xenograft model analysis also confirmed their positive relationship in vivo. In cell cycle analysis, consistent with findings of previous studies, NDRG1 overexpression induced G0/G1 arrest. p21 is the key regulator of G0/G1 arrest. This phenomenon was then restored through inhibiting p21 expression by si-RNA, and a similar “rescue” phenomenon was also observed in other proliferation-related assays such as CCK-8 and colony formation assays. Hence, NDRG1 may play a key role in inhibiting proliferation through up-regulating p21 expression.

There have been handful studies reported that NDRG1 can increase p21 protein expression in other diseases [25, 47]. However, the underlying mechanisms are still obscure. Given that p21 is not stable in vivo, posttranslational modification (PTM) is a key regulatory mechanism for maintaining its steady-state level [48]. Ubiquitylation is a critical PTM, which plays a significant role in all aspects of cell physiology and pathology, and the function of p21 is closely regulated by ubiquitylation [49]. Ubiquitylation by several E3 ligases is the major regulatory mechanism of p21 protein [50]. There are mainly three E3 ubiquitin ligase complexes responsible for ubiquitin-mediated degradation of p21: CRL4^CDT2^, SCF^SKP2^, and APC/C^CDC20^. CRL4^CDT2^ [51] specifically induces p21 ubiquitylation and degradation at the S phase of cell cycle, while SCF^SKP2^ [48] promotes p21 degradation in both G1/S transition and S phase. APC/C^CDC20^ [52] is reported to drive p21 degradation during mitosis. In addition, other E3 ligases such as FBXO22 [53], CHIP [54], SPSB1 [55], and RNF126 [56] can target p21 for ubiquitin-mediated degradation. This study showed that NDRG1 overexpression could suppress p21 ubiquitylation, while NDRG1 knockdown increased ubiquitylation of p21. After screening in UbiBrowser Databas, we matched 20 overlapped E3 ligases that may simultaneously interact with both NDRG1 and p21, among which NEDD4 was selected. NEDD4, also known as neural precursor cell expressed developmentally downregulated protein 4, has been reported as E3 ligase for many substrates, including EGFR, PTEN, and ERBB4, and it was involved in tumor and other diseases [57–59]. Not surprisingly, co-IP results showed that p21 could interact with NEDD4. p21 expression was up-regulated after si-NEDD4, and NEDD4 overexpression could increase the ubiquitylation of p21, indicating that p21 might be the substrate of NEDD4. Verma noted that NDRG1 could interfere with the combination between NEDD4 and its substrate in breast cancer [35]. Further experiments showed that NDRG1 overexpression could inhibit combination between NEDD4 and p21, therefore, suppressing p21 ubiquitylation and increasing its protein expression. Consistent with another study [47], our results indicated that the nuclear expression of p21 was significantly increased after NDRG1 overexpression, while NDRG1 knockdown decreased its nuclear expression, i.e., p21 translocation, by which NEDD4 was likely to interact with more cytoplasmic p21 and then induced p21 degradation.

As ubiquitylation is a complicated cascade, it’s obvious that NDRG1/NEDD4 cannot be the only factor to regulate p21 stability and ubiquitylation. In addition to E3 ligases we mentioned above, deubiquitylation by deubiquitylases is also indispensable for dynamic balance of p21 between stabilization and degradation. The study about deubiquitylation has been an emerging research field recently. Deng [50] reported that USP11 could directly remove p21 polyubiquitylation and protect p21 from ubiquitin-mediated degradation. Cables1 could stabilize p21 by inhibiting PSMA3-mediated proteasomal degradation [60]. Further studies are needed to clarify whether NDRG1 can regulate the functions of other E3 ligases and deubiquitylases. In this study, we showed that p21 could be the substrate of NEDD4 in CRC cell lines, added that NDRG1 was involved in the complex network regulating the p21 expression.

## Conclusions

In conclusion, our results demonstrated that NDRG1 could inhibit tumor growth in vivo and in vitro through increasing p21 protein expression by suppressing its ubiquitylation. NEDD4-mediated p21 degradation was inhibited because of NDRG1 overexpression, which may offer a new strategy for targeted therapy in CRC patients.

## Supplementary information

**Additional file 1: Table S1.** Twenty overlapped E3 ligases which may simultaneously interact with both NDRG1 and p21.

**Additional file 2: Figure S1.** Regulation of NDRG1 on p21 expression in both protein and mRNA levels. (A) Subcellular Fractionation Assay: expression of NDRG1 and p21 protein were detected in both nucleus and cytoplasm after NDRG1 overexpressed/silenced. Histone H3 and GAPDH were used as loading control for nucleus and cytoplasm, respectively. (B) qPCR analysis of NDRG1 and p21 mRNA levels after NDRG1 overexpressed/silenced. Error bar represents the mean ± SD of 3 independent experiments. ****p < 0.0001. *n.s* not significant.

**Additional file 3: Figure S2.** NEDD4 could promote the ubiquitylation of p21 and induce p21 degradation. (A) Plasmids of Flag-p21 and NEDD4 were co-expressed in 293 T cells (293 T Flag-p21/NEDD4). Co-immunoprecipitation were performed to confirm their interaction. (B) NEDD4 overexpression could decrease p21 protein level. (C) We overexpressed HA-Ubiquitin, Flag-p21 or NEDD4 in 293 T cells. Flag antibody was used to immunoprecipitate Flag-p21. p21 ubiquitylation was then detected by immunoblotting by ubiquitin antibody.

**Additional file 4: Figure S3.** The percentage of Ki-67-positive cells were quantified in Vector Control, NDRG1, sh-Control and SH-NDRG1 groups of Fig. 6d.

## Data Availability

All data presented or analyzed in this study are included either in this article or in the additional files.

## Abbreviations

CRC: Colorectal cancer
NDRG1: N-myc downstream regulated gene-1
OSCC: Oral squamous cell carcinoma
