# Supplementary figures and images for: N-myc downstream-regulated gene 1 inhibits the proliferation of colorectal cancer through emulative antagonizing NEDD4-mediated ubiquitylation of p21
Source: J Exp Clin Cancer Res. 2019 Dec 12;38:490. doi: 10.1186/s13046-019-1476-5 (PMC6909641; doi:10.1186/s13046-019-1476-5)

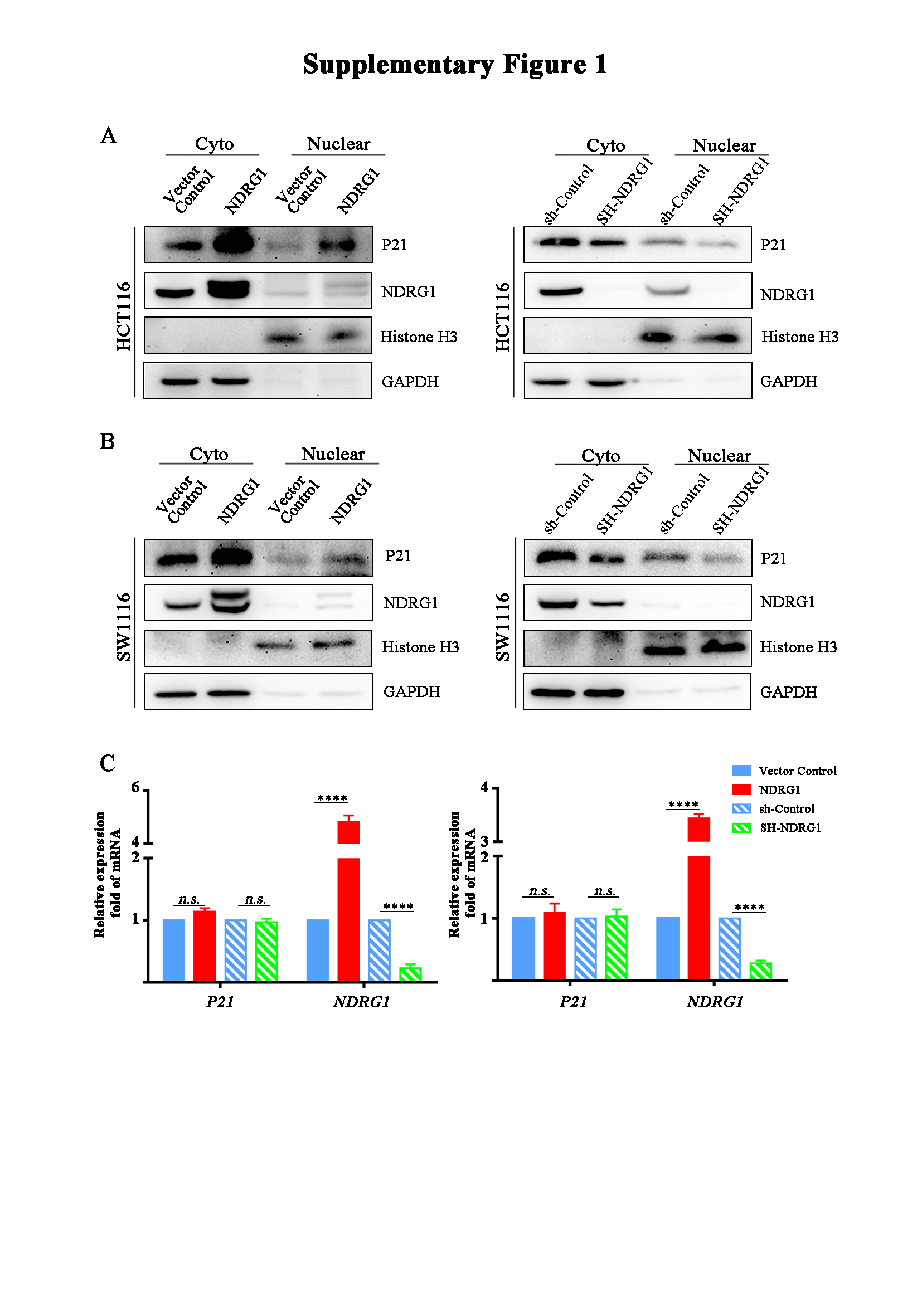

Supplement: Supplementary file 2 — Additional file 2: Figure S1. Regulation of NDRG1 on p21 expression in both protein and mRNA levels. (A) Subcellular Fractionation Assay: expression of NDRG1 and p21 protein were detected in both nucleus and cytoplasm after NDRG1 overexpressed/silenced. Histone H3 and GAPDH were used as loading control for nucleus and cytoplasm, respectively. (B) qPCR analysis of NDRG1 and p21 mRNA levels after NDRG1 overexpressed/silenced. Error bar represents the mean ± SD of 3 independent experiments. ****p < 0.0001. n.s not significant. [file 13046_2019_1476_MOESM2_ESM.tif]

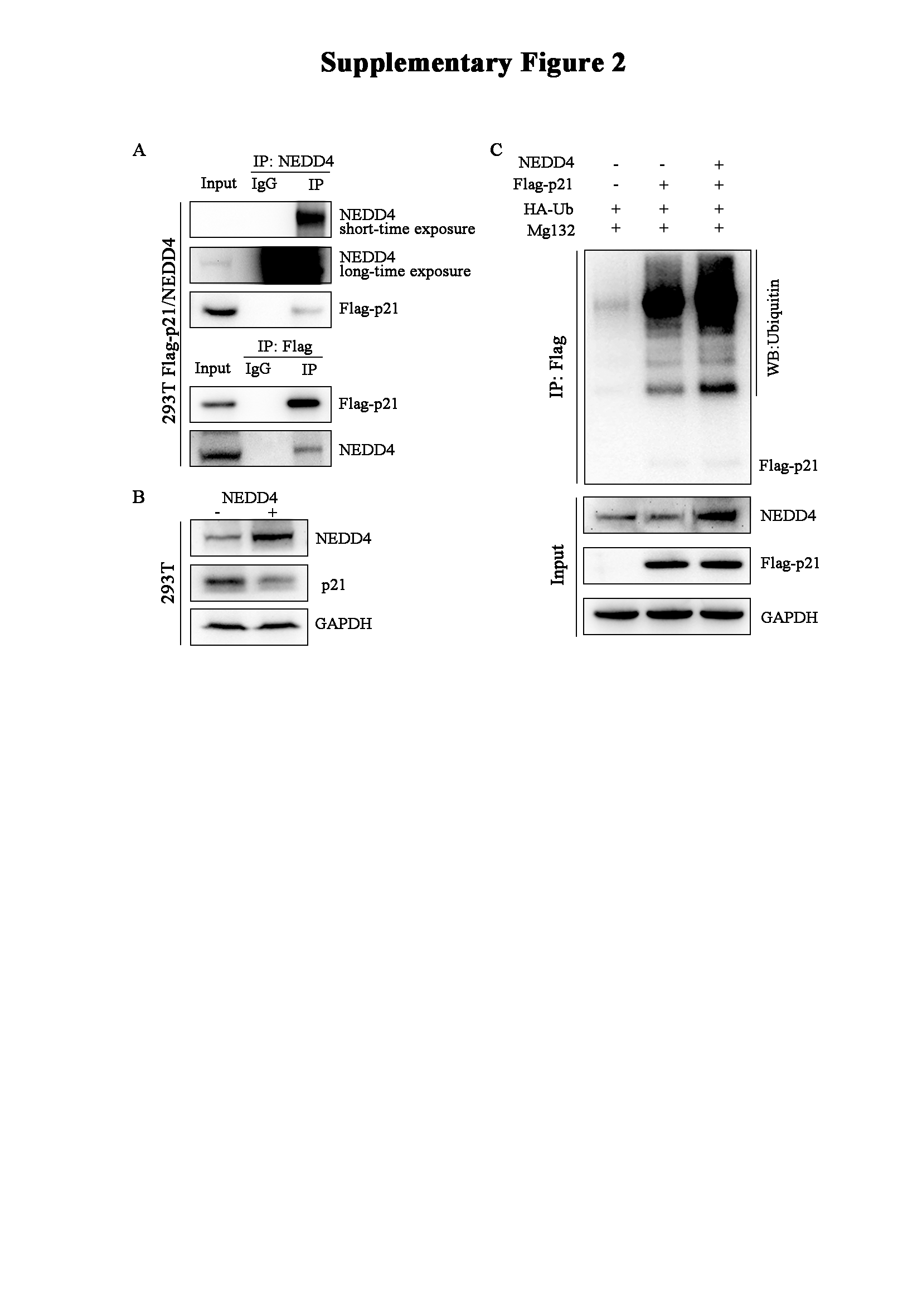

Supplement: Supplementary file 3 — Additional file 3: Figure S2. NEDD4 could promote the ubiquitylation of p21 and induce p21 degradation. (A) Plasmids of Flag-p21 and NEDD4 were co-expressed in 293 T cells (293 T Flag-p21/NEDD4). Co-immunoprecipitation were performed to confirm their interaction. (B) NEDD4 overexpression could decrease p21 protein level. (C) We overexpressed HA-Ubiquitin, Flag-p21 or NEDD4 in 293 T cells. Flag antibody was used to immunoprecipitate Flag-p21. p21 ubiquitylation was then detected by immunoblotting by ubiquitin antibody. [file 13046_2019_1476_MOESM3_ESM.tif]

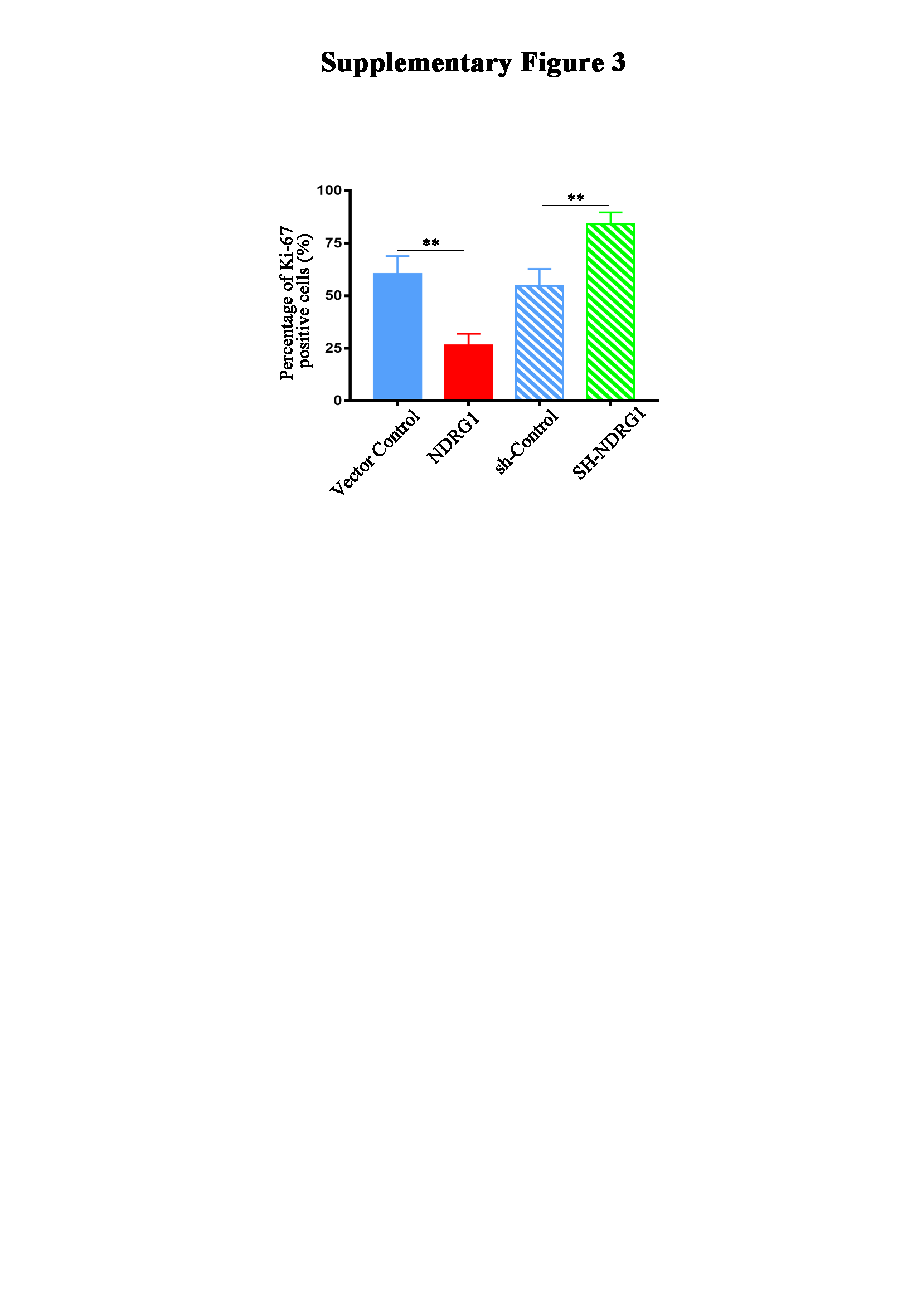

Supplement: Supplementary file 4 — Additional file 4: Figure S3. The percentage of Ki-67-positive cells were quantified in Vector Control, NDRG1, sh-Control and SH-NDRG1 groups of Fig. 6d. [file 13046_2019_1476_MOESM4_ESM.tif]
